# Supplementary material for: N6-methyladenosine modification of HIV-1 RNA suppresses type-I interferon induction in differentiated monocytic cells and primary macrophages
Source: PLoS Pathog. 2021 Mar 10;17(3):e1009421. doi: 10.1371/journal.ppat.1009421 (PMC7984636; doi:10.1371/journal.ppat.1009421)
Supplement: Attachment — Submitted filename: Authors responses to reviews 1-18-21.pdf [file ppat.1009421.s001.pdf]

Authors' responses to the reviewers' questions and comments

Manuscript #: PPATHOGENS-D-20-02166

Previous title: *N*^{6}-methyladenosine modification of HIV-1 RNA evades RIG-I-mediated sensing to suppresses type-I interferon induction in monocytic cells

New title: *N*^{6}-methyladenosine modification of HIV-1 RNA suppresses type-I interferon induction in differentiated monocytic cells and primary macrophages

Part I – Summary

Please use this section to discuss strengths/weaknesses of study, novelty/significance, general execution and scholarship.

Reviewer #1: In this original research article, Chen et al. look to test the hypothesis that m6A modification of the HIV RNA genome modulate the innate immune responses by inhibition of RNA sensing. Using a combination of in vitro RNA modifications and ex vivo gene editing approaches to alter m6A pathways, the authors show a very small, but consistent effect where less m6A correlates with more IFN induction in the monocytic cell line U937 and vice versa. Knockout of the sensors RIG-I and MDA5 suggest a reliance on RIG-I for sensing. Perhaps paradoxically to their argument, they find decreased levels of m6A modification in cellular RNA from virally suppressed patients on ART compared to viremic controls correlating with decreased IFN levels in vivo.

While the paper is generally well-written, with a clear hypothesis and a good attempt to bridge ex vivo and in vivo experimentation, the overall magnitude of the effect is not convincing, the reliance on one cell line model is limiting, the final mechanistic model is unclear, and several controls are missing that would improve interpretation of the results. Overall, while the results are consistent with a model in which m6A modification to RNA may provide limited protection against RIG-I sensing, it is unclear to what extent, if any, this is relevant to HIV infection. No effects on HIV infectivity are shown and the data collected in vivo seem at odds to their in vitro model. Several additional experiments are required to support the claims made in the paper.

Responses: We thank the reviewer for the positive review, constructive comments, and helpful suggestions. As the reviewer suggested, we have performed additional experiments and extensively revised the manuscript to further support our conclusions. Please refer to the detailed responses in Part II.

Reviewer #2: In this study, the authors showed that m6A modification of HIV-1 RNA suppresses the expression of IFN-I in human monocytic cell line U937. The authors demonstrated this effect of m6A via 1) transfection of cells with 42-mer RNA oligos (corresponding to sequences in HIV 5'UTR) that contain (or not) single m6A modification, 2) transfection of cells with RNA purified from virions with varying degrees of the m6A modification, and 3) infection of cells with HIV-1 containing different levels of the m6A modification. The authors further presented the data suggesting that the m6A modification allows HIV-1 to reduce activation of the transcription factors IRF3 and IRF7 that drive IFN-I gene expression and that m6A modification allows viral RNA to evade sensing by RIG-I but not MDA5. While the effects of changes in the m6A modification are modest in some experiments, the use of multiple methods to alter m6A modification levels makes the data convincing. These data are clearly presented and interpreted for the most part in a

balanced manner. As the finding that m6A modification of HIV-1 RNA promotes virus evasion of innate immune sensing is novel and potentially important, the manuscript is likely of interest to many readers of PLOS Pathogens. There are, however, a few points that the authors need to consider to improve the manuscript as described below.

Responses: We thank the reviewer for the positive review, constructive comments, and helpful suggestions. As the reviewer suggested, we have performed additional experiments and extensively revised the manuscript to further support our conclusions. Please refer to the detailed responses in Part II.

Reviewer #3: Chen and colleagues study the impact of HIV RNA m6A modifications on the induction of innate immune responses in monocytic cells. It has been recently found that m6A modifications can have an impact on RIG-I mediated infection in metapneumovirus, but similar studies have not been conducted with regards to HIV-1. The understanding of HIV-1 interactions with innate sensing machinery is very important to understanding HIV-1 pathogenesis, so this study is of high interest to both HIV researchers and general viral pathogenesis researchers. Using macrophage systems, the authors demonstrate the impact that m6A modification of the HIV-1 RNA can have on innate sensing. Transfection of differentiated monocytes with oligos harboring an m6A modification resulted in significantly reduced INF-alpha and –beta mRNA levels when compared to transfections using the same oligos without RNA alteration. The authors further support their hypothesis using a knockout of two different m6A eraser molecules driving a decrease in IFN-I responses. Interestingly, the same was observed when m6A was specifically inhibited by the compound DAA. In contrast, overexpression of the same erasers lead to the opposite effect. In addition, Chen et al. showed that infection with HIV RNA leads to phosphorylation/activation of IRF3 and IRF7, key regulators of the IFN-I pathways. The effect was even more pronounced when using conditions that increase m6A levels. In line with this, knockout of the RIG-I complex, a major component of the same pathway caused a significant decrease in IFN-I responses. Finally, the authors compared m6A levels and IFN-I responses in two HIV patient groups (viremic versus ART treatment) with a healthy control group. Both, m6A and IFN-I levels were highest in the viremic group and lowest in ART treated HIV patients.

Overall the quality of the data are high and the authors are careful to correlate the extent of m6A modification with the magnitude of the effects that they see. They present a robust data set in support of their hypothesis and their conclusions are sound. The size the effects that they measure are modest, but clearly affect IFN-I mRNA levels and IRF phosphorylation. It may be noted that the magnitude of the effect is weaker than strong agonists like poly IC, so it may be helpful to determine how vigorously this translates into differences in IFN-I protein levels or bioassay. Also although the impact of IFN-I on macrophage replication may not be expected to be large, it is worth commenting on whether this level of IFN-I induction has any affect on viral replication in monocytes (as I suspect this phenotype is more relevant to inflammation rather than direct effects on replication.) Lastly the patient data are intriguing, though an explanation for how the increase in m6A in patients relates to the monocyte cellular models, which indicate that elevated m6A should work to decrease inflammation, whilst it is clear that in vivo infection is associated with elevated IFN-I. This paper is of high interest.

Responses: We are encouraged by the reviewer's positive evaluation. Particularly, the reviewer highlighted that our data quality is high, our conclusions are sound, and our paper is of high interest. We appreciate the constructive comments and helpful suggestions from this reviewer. As the reviewer suggested, we have performed additional experiments and extensively revised the manuscript to further support our conclusions. Please refer to the detailed responses in Part II.

Part II – Major Issues: Key Experiments Required for Acceptance

Reviewer #1 Major issues:

1) Figure 1: While the oligo assay is clean and convenient, the relationship to HIV is tentative at best. While the 42-mers match HIV sequences, they are no substitute for incoming viral RNA or viral particles (tested directly in later figures). Rather, this figure seems to indicate a small (0.2 – 4.0 fold) effect on IFN induction by m6A modification in a sequence dependent manner. As the data is only shown normalized and without any positive or negative induction controls, it is unclear to what extent the oligos induce a response over baseline. Does m6A modification protect other oligos in random sequence? Would this difference be seen in other cell line models or in primary macrophages?

Responses: Thanks for the comments and questions. As the reviewer suggested, we have performed new experiments using a pair of RNA oligos in random sequence (scrambled oligo 2 with or without m^6A modification). To examine the extent the oligos induce a response over baseline, we also include the negative control of mock (no RNA oligo) transfection and the positive control of poly (I:C) in the transfection assay. Please refer to new results in Fig. 1F-G. These results indicated that IFN-I mRNA expression induced by m^6A-deficient RNA oligos is likely independent of RNA sequence. As reviewers suggested, we have also performed new experiments with primary macrophages transfected with HIV-1 RNA that had altered m^6A levels or control HIV-1 RNA (new Fig. 8A-F).

2) Figure 2: In vitro modification and subsequent transfection of HIV RNA results in 10-fold changes to m6A levels, but this has barely a 3-fold effect on IFN. HIV RNA from FTO overexpression cells similarly has 10-fold less m6A, but barely results in a 2-fold effect on IFN compared to a 200-fold effect of the positive control. Is there any proposed explanation for the differential scaling of these effects? Could this be driven by changes to RNA stability in the cell after delivery, which m6A has been shown to directly effect, rather than by direct protection from sensing?

Responses: Thanks for the reviewer's comments and insightful question. We also noticed the different effects on IFN-I induction between HIV-1 RNA and the positive control poly(I:C). Compared to poly(I:C) which mimics viral RNA, HIV-1 RNA induced lower levels of IFNs in cells likely due to the differences in RNA structures and pathways. Poly(I:C) is a synthetic analog of double-stranded RNA and is often recognized by endosomal TLR3 [1]. It also could activate the cytosolic RNA helicases RIG-I and MDA-5 [2]. We thus included the poly (I:C) controls in our studies (Fig. 1G, Fig. 8B-C, Fig. 9B, and Fig 10B). However, several cellular DNA and RNA sensors can play multifaceted roles in inducing innate immune responses to HIV-1 RNA and cDNA during different stages of the HIV-1 life cycle (reviewed in [3]).

It might be possible that changes to RNA stability in the cell can be affected by m^6A modification. However, it is important to note that other types of HIV-1 RNA modifications [4] can also play a role in avoiding intracellular sensing and IFN-I induction. For example, 2′-O-methylation in HIV-1 RNA prevents MDA5-mediated sensing in myeloid cells, and thereby reduces IFN-I induction [5]. To better explain our results, we have added the discussions of the results in the revised manuscript (page 14, last paragraph, lines 311-317).

3) Figure 2: This is further examined by infection with viruses produced from FTO overexpression cells and these show again a barely 2-fold increase in IFN over control viruses. Would a two-fold

effect on IFN influence HIV replication overall? What are the percent infected cells after this challenge was performed?

Responses: Thanks for your comments. We have performed the suggested experiments to examine the percent HIV-1-infected cells by intracellular staining of p24 and flow cytometry. We used the RT inhibitor nevirapine (NVP) to block HIV-1 infection. However, due to technical difficulty, we were not able to detect p24 staining that could be specifically inhibited by NVP. Alternatively, we detected HIV-1 *gag* mRNA in infected U937 cells and presented the results in new Fig. 4B in the revised manuscript.

4) Figure 2: The overall model that these data suggest is also somewhat puzzling as, to be sensed, the genomic RNA has to be released from the core at some point. Have the authors tried doing this experiment in the presence of an RT inhibitor to show that this is modified genomic RNA and not abortive RT transcripts driving this response?

Responses: We appreciate the insightful question. It is possible that the HIV-1 genomic RNA can be partially sensed in the infected cells during reverse transcription. To address the reviewer's question, we have performed HIV-1 infection experiment in the presence or absence of the RT inhibitor nevirapine. Our new data in Fig. 4 indicated that *IFN-I* mRNA induction by HIV-1 infection is not fully dependent on viral reverse transcription or productive infection, suggesting that m^6A-deficient HIV-1 genomic RNA triggers IFN-I induction in differentiated monocytic cells, at least in part.

5) Figure 3: Similar concerns with scale are raised as with Figure 2. For example, ALKBH5 overexpression decreases m6A levels by 50%, but has a similar impact on IFN as FTO overexpression, which reduces m6A levels by 90%? This would suggest that the impact on IFN does not directly correlate with m6A levels, but may be limited by some other factor.

Responses: We appreciate the comments and agree with the reviewer. We have added discussions regarding other types of modifications of HIV-1 RNA can also contribute to innate immune evasion (page 14, last paragraph, lines 311-317).

6) Figure 4: Knock-out of FTO and ALKBH5 look great, though the impact of ALKBH5 knock-out on HIV RNA m6A levels is greater than FTO, directly opposite the observation with overexpression. Transfection of this RNA shows a consistent though again minimal impact with up to 25-fold more m6A resulting in only a roughly 75% decrease in IFN induction. Parallel infection experiments show less than a 50% effect. This would be more convincing if the result could be rescued by treatment of the FTO-KO or ALKBH5-KO RNA with FTO in vitro. Still, the impact of m6A levels on RNA stability may serve as a significant confounder to these results.

Responses: We understand the reviewer's concern and appreciate the helpful suggestion. We have performed the suggested rescue experiment by treating the HIV-1 RNA from FTO-KO HEK293T cells with recombinant FTO in vitro and then transfecting the derived HIV-1 RNA into PMA-differentiated U937 cells. FTO treatment of HIV-1 RNA from FTO-KO cells reduced m^6A level approximately 3-fold, but the level was still 5.5-fold higher than that of control HIV-1 (Fig. R1A on the next page). Consistently, we observed reduced IFN-I induction in U937 cells transfected with HIV-1 RNA from FTO-KO cells compared to control HIV-1 RNA (Fig. R1B). However, FTO-treated HIV-1 RNA from FTO-KO cells did not show a significant change of IFN-I mRNA expression in U937 cells compared to HIV-1 RNA from FTO-KO cells (Fig. R1B). It is possible that more significant reduction of m^6A level by FTO treatment is required to obtain the rescue effect. To avoid confusion, we would not show the results in the revised manuscript.

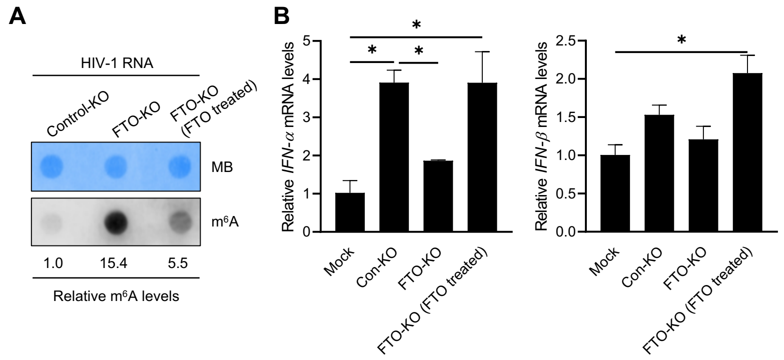

Figure R1. IFN-I mRNA expression in U937 cells transfected with HIV-1 RNA treated with recombinant FTO. (A) FTO treatment reduced the m^{6}A level of HIV-1 RNA (50 ng) from FTO-KO HEK293T cells. **(B)** The indicated HIV-1 RNA (250 ng) was transfected in PMA-differentiated U937 cells. At 16 hr post-transfection, *IFN-α* and *IFN-β* mRNA levels were measured by RT-qPCR. The results are shown as means ± S.D. of triplicated samples. * $P < 0.05$.

It might be possible that the impact of m^{6}A levels on RNA stability may serve as a potential confounder. We would like to address this important question in the future. However, it is also important to note that other types of HIV-1 RNA modifications [4] can also play a role in avoiding intracellular sensing and IFN-I induction. For example, 2′-O-methylation in HIV-1 RNA prevents MDA5-mediated sensing in myeloid cells, and thereby reduces IFN-I induction [5]. We have added discussions to help explain our results (page 14, last paragraph, lines 311-317).

7) Figure 5: The inhibitor is effective, but the IRF3 and IRF7 blots are nowhere near clear enough for precise quantification. The 1.2 and 1.7-fold effects are not convincing by these data.

Responses: We agree that the effects on IRF3 and IRF7 phosphorylation were small in the previous experiment. We have performed two new experiments and the results clearly showed increased phosphorylation of IRF3 and IRF7 (approximately 2- to 3-fold) in PMA-differentiated U973 cells that were infected with m^{6}A-deficient HIV-1 (derived from FTO- or ALKBH5 overexpressing HEK293T cells) compared to mock infection or normal HIV-1 derived from control HEK293T cells (new Fig. 3E and Fig. 5F).

8) Figures 1-5: At some point, the most pertinent results should be validated in primary cells.

Responses: We agree with the reviewer and we have performed new experiments using primary monocyte-derived macrophages. We presented the data in new Figure 8.

9) Figures 6 and 7: Though the phenotype is still small, the RIG-I dependency (rather than MDA5) is clear. It would be better to compare two KO lines as the shMDA5 line may have residual activity. These results are consistent with previous reports from Durbin et al. that m6A specifically protects against RIG-I sensing. My biggest concern here is again it is unclear how much these small oligos really reflect sensing of HIV RNA, nonetheless HIV RNA in the context of infection. One small note to the authors is they may want to validate these lines with Sendai virus infection as it is specifically sensed by RIG-I, but not as much by MDA5.

Responses: We agree that it is better to compare both RIG-I and MDA5 knockout cell lines as the shMDA5 line may have residual activity. Our shMDA5 cell line showed efficient (97% based on quantification of Western blotting of the specific band) knockdown of endogenous MDA5 (Fig. 10A). Unfortunately, we have not been able to generate a U973 single-cell clone with complete MDA5 knockout, which requires additional time and efforts. We understand the concern about whether the small oligos can fully reflect sensing of HIV-1 RNA in the context of infection. We

would like to address this question with HIV-1 infection and HIV-1 RNA transfection when we generate the MDA5 knockout U937 cell line in the future.

We appreciate the kind suggestion on Sendai virus infection and we will include the control when we establish the MDA5 knockout cell line. We included the positive control of poly (I:C) transfection to verify the significantly reduced RNA sensing ability in RIG-I KO or MDA5 KD cells (Fig. 9B and Fig. 10B, respectively).

10) Figure 8: While this effort to gather in vivo data is appreciated, it is unclear how monitoring overall levels of cellular RNA m6A in control, viremic, and ART patients contributes to the overall narrative of the manuscript. While the data show statistically significant differences in m6A levels between viremic and ART patients, the number of patients is too small to make any definitive conclusions or to meaningfully control for any demographic or virologic confounders. IFN levels in viremic individuals is higher than control or ART patients, consistent with prior reports, but this almost contradicts the model that more m6A protects against IFN induction.

Responses: We appreciate the comment and agree with the reviewer's suggestion. To avoid confusion and confounding results due to small patient sample size, we have removed previous Fig. 8 and related text of the HIV-1 patient samples. We will obtain more patient samples to perform more experiments and analyses in a future study.

Reviewer #2: Major issues:

1. The patient-based data shown in Fig 8 are unrelated to the rest of the study described in the manuscript. This figure compares the average m6A level in total RNA of PBMCs from HIV-1 viremic patients with that from patients on ART. As the main focus of the manuscript is m6A modification of HIV-1 RNA, not the total cellular RNA, this part is irrelevant to the overall conclusion of the manuscript in the current form and thus should be removed.

Responses: We appreciate the comment. We agree with the reviewer that the result of patient sample is irrelevant to the overall conclusion of the manuscript. We have removed previous Fig. 8 and related text of the HIV-1 patient samples. We will obtain more patient samples to perform more experiments and analyses in a future study.

2. The conclusion that m6A modification allows viral RNA to evade RNA sensing by RIG-I is based on the results obtained with transfection of the 42-mer RNA oligos (Figs 6 and 7). This should be confirmed through infection of cells with HIV-1 containing different levels of m6A modification as done in Figs 2-5.

Responses: We appreciate the comment and helpful suggestion. We agree with the reviewer that HIV-1 infection of U937 cells with RIG-I or MDA5 is important to confirm the observation using HIV-1 RNA oligos. We would like to perform HIV-1 infection assays when we establish the MDA5 knockout U937 cell line, which requires significant more time and effort.

Furthermore, to better reflect our new results and not to emphasize the conclusion on RIG-I-mediated sensing of m^6A-defective HIV-1 RNA, we have changed the title of the revised manuscript to: "N^6-methyladenosine modification of HIV-1 RNA suppresses type-I interferon induction in differentiated monocytic cells and primary macrophages". We have also revised the abstract, author summary and main text accordingly.

3. All presented data (except for Fig 8) were obtained using a monocytic cell line U937. It would be ideal to confirm at least key parts of the results using primary monocytes or monocyte-derived APCs.

Responses: We agree with the reviewer and appreciate the suggestion. We have performed experiments with primary monocyte-derived macrophages. Please see new results in Fig. 8A-F.

Reviewer #3: Major issues:

1) In differentiated monocytes the authors show that transfection/infection of HIV RNA with more m6A inversely correlates with IFN-I responses. They also come up with a model describing that m6A seems to mask HIV RNA from being recognized by the RNA sensing pathway in the host cell (Sup. Figure S2). However, in patients they show a positive correlation between m6A levels and IFN-I responses (the viremic group has highest levels of m6A and IFN-I). How does this fit into their in vitro model? This seems inconsistent with their in vitro model.

Responses: We appreciate the comment. We agree with the reviewer that the result of patient sample is irrelevant to the overall conclusion of the manuscript. We have removed previous Fig. 8 and related text of the HIV-1 patient samples. We have also added new results derived from primary macrophages (new Fig. 8).

2) In their study, Chen et al measured all their IFN-I responses on RNA level. These also correlated with IRF phosphorylation, so this is promising. To demonstrate that there is an actual increase on the IFN protein level and/or a bioassay would be further compelling to demonstrate functional significance of the IFN-I mRNA levels.

Responses: Thanks for the suggestion. We have performed ELISA to detect IFN-I protein levels in the supernatants of primary macrophages that were transfected with HIV-1 RNA containing altered m^6A levels compared to control HIV-1 RNA. We included these new results in new Fig. 8C and 8E.

3) In the last line of the discussion the authors suggest that antagonism of m6A modification can lead to increased IFN-I and decreased replication. Does it have an impact on replication? A discussion of how IFN-I may be expected to influence replication would help put this into context.

Responses: Thanks for the comments. We have performed the HIV-1 infection assay and added new results (Fig. 4). We have also revised the discussions based on the new data.

Part III – Minor Issues: Editorial and Data Presentation Modifications

Reviewer #1: (No Response)
Reviewer #2: (No Response)

Reviewer #3: Minor issues:

1) Page 10/11, lines 225-234: As a matter of interpretation: The authors describe in the MDA5 knockdown experiments with subsequent HIV RNA transfection/infection that they don't see a full abrogated IFN-I response (Figure 5) as with knockdown of RIG-I and draw the conclusion that MDA5 does not contribute to the recognition of HIV RNA. Is it possible that the difference in m6A

vs. non m6A induced IFN-I response in the knockdown experiment is due to the residual MDA5? Figure 7A shows that there is not a total knockdown of MDA5 as compared to RIG-I.

Responses: We understand the concern. Our stable shMDA5 U937 cell line showed a 97% reduction of endogenous MDA5 expression (Fig. 10A). However, we have not been able to generate a single U937 cell clone with MDA5 knockout, which would require additional time and efforts. We would like to confirm the genome editing of the *MDA5* gene in U937 cells by DNA sequencing, but our sequencing core facility is currently not in full service due to the COVID-19 pandemic. We hope that the reviewer understands our situation and plan.

2) Page 11, line 245: There is no inverse correlation, but a positive correlation of m6A with viral load.

Response: As the reviewers suggested, we have removed previous Fig. 8 and related text of the HIV-1 patient samples.

3) Page 12, line 256: There is no significant decrease comparing the HIV viremic and ART group for IFN-alpha, only for IFN-beta.

Response: As the reviewers suggested, we have removed previous Fig. 8 and related text of the HIV-1 patient samples.

4) Figure 8A-C: Is there a correlation of viral load with m6A levels/IFN-alpha/IFN-beta levels within the viremic HIV group? A separate graph showing a correlation would be helpful.

Response: We appreciate the suggestion. As the reviewers suggested, we have removed previous Fig. 8 and related text of the HIV-1 patient samples. We will obtain more patient samples to complete the experiments and analysis in a future study.

5) We would suggest incorporating Sup Fig. S2 into the text.

Response: We changed Fig. S2 to Fig. 11 into the main text as suggested.

6) Page 14, line 302: mice

Response: We changed it as suggested.

7) Page 14, line 312: modifications

Response: We changed it as suggested.

8) Page 14, line 316: reduce

Response: We changed it as suggested.

References cited in the responses:

1. Alexopoulou L, Holt AC, Medzhitov R, Flavell RA. Recognition of double-stranded RNA and activation of NF-kappaB by Toll-like receptor 3. Nature. 2001;413(6857):732-8. Epub 2001/10/19. doi: 10.1038/35099560. PubMed PMID: 11607032.

2. Kato H, Takeuchi O, Sato S, Yoneyama M, Yamamoto M, Matsui K, et al. Differential roles of MDA5 and RIG-I helicases in the recognition of RNA viruses. Nature. 2006;441(7089):101-5. Epub 2006/04/21. doi: 10.1038/nature04734. PubMed PMID: 16625202.

3. Yin X, Langer S, Zhang Z, Herbert KM, Yoh S, Konig R, et al. Sensor Sensibility-HIV-1 and the Innate Immune Response. Cells. 2020;9(1). Epub 2020/01/24. doi: 10.3390/cells9010254. PubMed PMID: 31968566; PubMed Central PMCID: PMCPMC7016969.

4. Courtney DG, Tsai K, Bogerd HP, Kennedy EM, Law BA, Emery A, et al. Epitranscriptomic Addition of m(5)C to HIV-1 Transcripts Regulates Viral Gene Expression. Cell Host Microbe. 2019;26(2):217-27 e6. doi: 10.1016/j.chom.2019.07.005. PubMed PMID: 31415754; PubMed Central PMCID: PMCPMC6714563.

5. Ringeard M, Marchand V, Decroly E, Motorin Y, Bennasser Y. FTSJ3 is an RNA 2'-O-methyltransferase recruited by HIV to avoid innate immune sensing. Nature. 2019;565(7740):500-4. doi: 10.1038/s41586-018-0841-4. PubMed PMID: 30626973.
