# Supplementary material for: N6-methyladenosine modification of HIV-1 RNA suppresses type-I interferon induction in differentiated monocytic cells and primary macrophages
Source: PLoS Pathog. 2021 Mar 10;17(3):e1009421. doi: 10.1371/journal.ppat.1009421 (PMC7984636; doi:10.1371/journal.ppat.1009421)
Supplement: Attachment — Submitted filename: Author Responses to Reviewer comments 2-20-21.pdf [file ppat.1009421.s002.pdf]

Authors' Responses to the Reviewers' Questions and Comments

February 20, 2021

Manuscript #: PPATHOGENS-D-20-02166R1

Title: N^6-methyladenosine modification of HIV-1 RNA suppresses type-I interferon induction in differentiated monocytic cells and primary macrophages

Editors' Comments:

The reviewers appreciated the attention to an important topic. Based on the reviews, we are likely to accept this manuscript for publication, providing that you modify the manuscript according to the review recommendations.

Although all of the reviewers felt that your revised manuscript is greatly improved, Review 1 still has a few remaining concerns. As an opportunity to use these comments to further improve your study, I would ask that you respond to them to the extent that you feel is constructive.

Responses: We are pleased that the three reviewers appreciated the attention to an important topic, and that all of the reviewers felt that our revised manuscript is greatly improved. We have made our very best effort to address questions of reviewer #1 and further improve our work. We have performed key experiments suggested by the reviewer. Our specific responses are the following:

Part I – Summary
Please use this section to discuss strengths/weaknesses of study, novelty/significance, general execution and scholarship.

Reviewer #1: In this original research article, Chen et al. look to test the hypothesis that m6A modification of the HIV RNA genome modulates the innate immune response to infection by inhibition of RNA sensing. Using a combination of in vitro RNA modifications and ex vivo gene editing approaches to alter m6A pathways, the authors show a small, but consistent effect where less m6A correlates with more IFN induction in the monocytic cell line U937 and vice versa. This phenotype is RIG-I dependent and further correlates with phosphorylation of IRF3/7. Important aspects of the phenotype are validated in primary MDMs.

This manuscript reflects a major improvement over the first submission. I would like to commend the authors for their hard work in responding to my comments, which I know were extensive. A majority of my concerns were addressed. The disconnect between the magnitude of the changes in m6A levels versus the magnitude of effect on IFN induction is never really addressed, but I can accept that this is something for future work. However, there is still one critical control that I think must be addressed prior to publication as it directly influences how these data are interpreted, namely viral RNA quantification before challenge (see Major Issues). Besides that, I only have a couple of minor suggestions.

Responses: We appreciate that the reviewer recognized our best efforts in responding to previous comments. We also appreciate the reviewer's positive evaluations and constructive comments. Our point-by-point responses to the reviewer's questions are detailed in part II on the next page.

Reviewer #2: In this revision, the authors added a substantial amount of new data, which provided supporting evidence for the physiological significance of the original observations and further advanced mechanistic understanding. Removing the in vivo data has also helped clarify the focus of the study. Overall, in my opinion, the authors have satisfactorily addressed the concerns raised by the reviewers in the previous round.

Responses: We appreciate the reviewer's very supportive comments.

Reviewer #3: A revised manuscript from Chen et al has made extensive changes to address the questions raised in the initial review. The study is thorough in looking at the activation of type I IFN responses by RNA that is hyper or hypo modified by N6-methyladenosine. The most significant changes in the manuscript are the removal of the clinical data, which did not connect well with the observations made in cell lines. And the addition of very relevant data set regarding the response of primary monocyte derived macrophages (MDM) to changes in m6A. The studies in MDM show robust effects and are considerably more relevant to normal physiology of HIV and the induction of inflammatory responses. The authors find that m6A modification suppresses IFN induction in primary MDM and also find that strong enhancement of IFN induction when erasers are used to remove m6A modification. The modifications have strengthened the manuscript substantially.

Responses: We appreciate the reviewer's very supportive comments.

Part II – Major Issues: Key Experiments Required for Acceptance
Please use this section to detail the key new experiments or modifications of existing experiments that should be <u>absolutely</u> required to validate study conclusions.

Reviewer #1: Figures 3-8 all rely on an assay wherein HIV-1 is harvested from culture supernatants and subsequently used for downstream challenge, either directly or using extracted RNA. In most cases, the authors show that the cells express similar levels of p24, that different conditions have similar levels of total RNA, and that the expected changes in m6A levels are observed. A critical missing component, however, is how much live virus or viral RNA is contained in those samples. As the changes in IFN induction are only 2-3 fold in most cases, small differences in the amount of virus produced or the amount of viral RNA packaged could drastically influence the downstream result. Given the new data in Figure 4B (that there is 30-fold more HIV-1 gag RNA produced after challenge of U937 cells with virus from FTO o/e cells), this information is even more critical. Is altering FTO or m6A pathways in producer cells influencing downstream IFN sensing in target cells primarily by influencing the amount of viral RNA produced or packaged? If you DNase treat the RNA in Figure 3C, for example, and do qPCR for viral RNA, is it equal? I think this is really important for interpretation of the phenotype (and may also speak to the magnitude issue mentioned above… I suspect that most of the RNA extracted is not actually

viral RNA, but cellular RNA from exosomes isolated alongside the VLPs. All of this RNA may show changes in m6A levels, but it might not all be sensed like viral RNA is.).

Responses: We appreciate the reviewer's thoughtful questions and comments. We would like to address three major questions raised by the reviewer as follows:

1) We agree that it is important to know how much live HIV-1 or viral RNA is contained in the samples used in HIV-1 infection and viral RNA transfection assays (previous Fig. 3-8). To address reviewer's question, we examined the infectivity of HIV-1 generated from HEK293T cells overexpressing FTO compared to control HIV-1 using a reporter cell line (HeLa-derived TZM-bl cells). Our new results show comparable infectivity between these two viral stocks based on the same amounts of p24 input (new Fig. 3D).

   Furthermore, we used the same amounts of HIV-1 p24 capsid to normalize HIV-1 input for infection because we have published the quantification data of HIV-1 RNA m^6A levels using mass-spectrometry (Tirumuru et al. *eLife,* 2016; PMID: 27371828). Please refer to the following data and figure from the *eLife* paper, showing consistent m^6A levels in HIV-1 RNA between two different virus preparations.

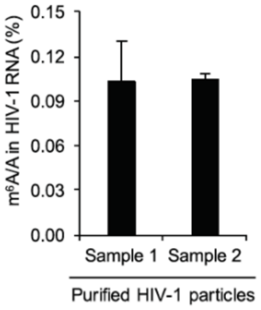

   This figure is from our previous paper (Tirumuru et al. *eLife,* 2016).

   Figure 1—figure supplement 2. Quantification of HIV-1 RNA m^6A level using liquid chromatography-mass spectrometry. HIV-1 RNA (250 ng) was isolated from highly purified HIV-1_{MN} virions (total 600 mg of p24 capsid) and subjected to quantitative analysis of the m6A level using LC-MS/MS (n=3 of each sample). The results are presented are from representative of two independent experiments.

   In this study, we also quantified m^6A levels in HIV-1 RNA using our optimized m^6A dot-blot assay (Tirumuru et al. *JBC,* 2019; PMID: 30617182), which includes methylene blue staining of RNA as a loading control for better quantification. Therefore, we are confident about quantification of m^6A levels in HIV-1 RNA showed in experiments in revised Fig. 3-9.

2) Is altering FTO or m^6A pathways in producer cells influencing downstream IFN sensing in target cells primarily by influencing the amount of viral RNA produced or packaged?

   This is an interesting question that we have not directly addressed. In the manuscript, however, we showed that overexpression or knockout of FTO or ALKBH5 in virus producer HEK293T cells did not significantly affect intracellular HIV-1 Gag and p24 expression (Fig. 3B, 5B and 7B). Treatment of HEK293T cells with the m^6A inhibitor DAA did not affect HIV-1 production and release (Fig. 6B). Because HIV-1 RNA package is dependent on its interaction with Gag, these results would suggest that the amount of HIV-1 RNA produced or packaged likely are not affected by altering FTO or m^6A pathways in producer cells. Moreover, for RNA transfection assays, we quantified and used the same amounts of HIV-1 RNA isolated from purified virions.

3) If you DNase treat the RNA in Figure 3C, for example, and do qPCR for viral RNA, is it equal? I think this is really important for interpretation of the phenotype.

As we described in Materials and Methods of our manuscript, HIV-1 pellet was resuspended with PBS and digested with DNase I (Turbo, Invitrogen) for 30 min at 37 °C to remove any plasmid DNA used in transfection of HEK293T cells to produce HIV-1. To extract HIV-1 genome RNA, concentrated HIV-1 virions were lysed by Trizol (Invitrogen) and RNA was purified by phenolic-chloroform sedimentation and isopropanol precipitation. Because we used purified HIV-1 RNA in our transfection experiments (Fig. 3C and others figures showing HIV-1 RNA transfection results), we are confident about our results and interpretation of the phenotype.

Reviewer #2: I have no more concerns.
Reviewer #3: No major issues noted.

Responses: We thank reviewers #2 and #3 for their support.

Part III – Minor Issues: Editorial and Data Presentation Modifications

Please use this section for editorial suggestions as well as relatively minor modifications of existing data that would enhance clarity.

Reviewer #1:

1) I would include a paragraph in the introduction talking more about previous molecular studies that show the importance of the m6A pathway in HIV-1 replication.

Responses: We appreciate the reviewer's suggestion. We have added a paragraph in the revised introduction (the second paragraph, lines 75-88 on page 4) to highlight the importance of the m^6A pathway in HIV-1 replication.

2) Figure 8 shows minimal impacts on m6A after FTO-OE, but huge impacts on sensing. If only some sites in the genome are important for sensing, m6A-Seq may be very informative here. I know this is outside the scope, but it would be really cool to do.

Responses: We appreciate the reviewer's suggestion. Based on our results in previous Figure 8 (Figure 9 in this revision), we think that primary macrophages are more sensitive than PMA-differentiated U937 cells in sensing m^6A-defective HIV-1 RNA. We agree with the reviewer that it is informative to identify the specific m^6A sites in the HIV-1 genome that are important for sensing. In fact, this is one of specific aims that we designed in our pending grant application to continue this project. We plan to address this important question using m^6A-Seq in combination with mutagenesis and functional studies. We also agree with the reviewer that this question is outside the scope of current study. Thus, we plan to answer this question and publish the results in a separate manuscript in the future.

3) I actually find the rescue data that you shared very compelling! As discussed above, I would bet that slight discrepancies in the amount of viral RNA may explain some of the fluctuation. If you have a chance to repeat it, it might trend towards a significant difference at which point I would highly recommend including it in the manuscript.

Responses: We are pleased that reviewer found the rescue data very compelling, which we showed in our previous responses to reviewers' questions. We appreciate the reviewer's suggestion to include the data in the manuscript. We have repeated the rescue experiments and included the data in new Figure 8. We agree with the reviewer that these new data can further support our conclusions and strengthen our manuscript.

Reviewer #2: (No Response)

Reviewer #3: Might add a bit of introductory background regarding the role of monocytes in pathogen sensing and inflammation in HIV. This bit of additional context in the intro may add to the significance of the study.

Responses: We appreciate the reviewer's suggestion. Accordingly, we have added more introductory background regarding the role of monocytes in pathogen sensing and inflammation in HIV-1 (see line 101-104 in the second last paragraph of introduction on page 5).
